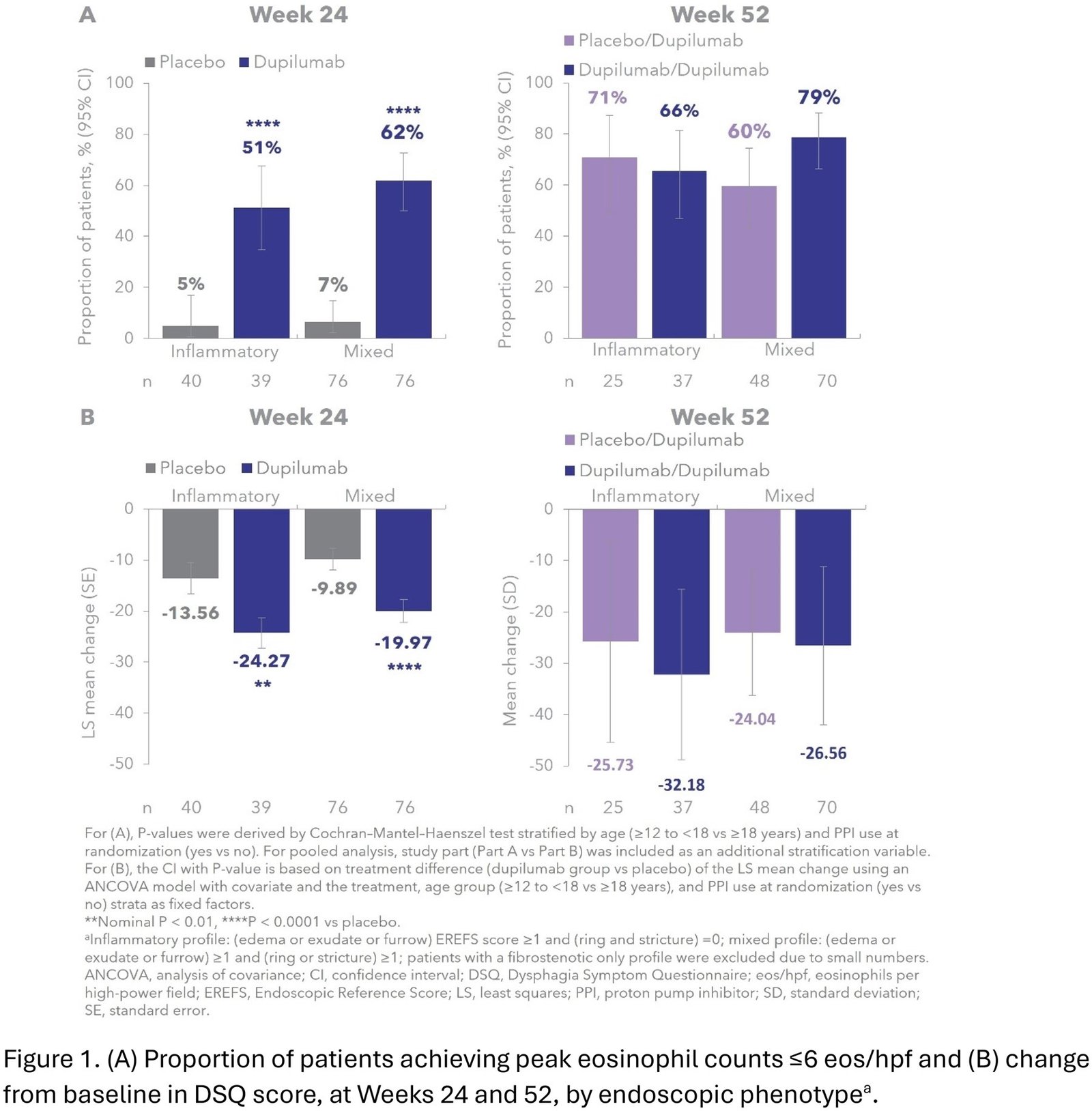# Poster Session II - A192 DUPILUMAB IMPROVES FEATURES OF EOSINOPHILIC ESOPHAGITIS REGARDLESS OF ENDOSCOPIC PHENOTYPE: LIBERTY EOE TREET STUDY POST HOC ANALYSIS

**DOI:** 10.1093/jcag/gwaf042.192

**Published:** 2026-02-13

**Authors:** E V Savarino, K Peterson, M H Collins, R A Pollock, C Xia, S Zaghloul, B Raphael, J T Angello, A Radwan

**Affiliations:** University Hospital of Padova, Padua, Italy; University of Utah, Salt Lake City, UT; Cincinnati Children’s Hospital Medical Center and University of Cincinnati College of Medicine, Cincinnati, OH; Sanofi, Toronto, ON, Canada; Regeneron Pharmaceuticals Inc., Tarrytown, NY; Sanofi, Bridgewater, NJ; Regeneron Pharmaceuticals Inc., Tarrytown, NY; Sanofi, Bridgewater, NJ; Regeneron Pharmaceuticals Inc., Tarrytown, NY

## Abstract

**Background:**

In LIBERTY EoE TREET (NCT03633617), dupilumab improved histologic, symptomatic, and endoscopic EoE features up to 52 weeks.

**Aims:**

To assess the effect of dupilumab in adolescents and adults with EoE by baseline endoscopic profile.

**Methods:**

Patients (pts) with EoE aged ≥12 years received either 24 weeks of dupilumab 300 mg weekly or placebo (PBO). Eligible pts who completed 24 weeks of treatment received dupilumab to Week (W)52. Pts were categorized by baseline endoscopic profile (inflammatory or mixed). Pts achieving peak eosinophil count (PEC) ≤6 eosinophils per high-power field (eos/hpf) and change from baseline in Dysphagia Symptom Questionnaire (DSQ) score, Endoscopic Reference Score (EREFS) total score, inflammatory (edema, exudate, furrow) and fibrostenotic (ring, stricture) subscores were assessed at W24 and W52. All *P*-values are nominal.

**Results:**

Of 240 pts, 32.9%/63.3% had an inflammatory/mixed baseline endoscopic profile. Pts with a mixed profile were older than those with an inflammatory profile (mean 33.8 years [standard deviation 12.4] vs 20.7 [10.9], *P*<0.0001). At W24, more pts achieved PEC ≤6 eos/hpf with dupilumab vs PBO, regardless of endoscopic profile (inflammatory: 50.5% difference [95% confidence interval (CI) 32.1, 68.9]; mixed: 57.4% [44.8, 70.1]; both *P*<0.0001) (Fig 1A). Dupilumab improved DSQ score vs PBO at W24, regardless of endoscopic profile (least squares mean difference [LSMD] [95% CI] −10.7 [−18.7, −2.7], *P*<0.01; −10.1 [−14.9, −5.3], *P*<0.0001) (Fig 1B). At W52, further improvements were observed in pts who continued dupilumab; improvements were also reported in pts who switched from PBO to dupilumab at W24 (Fig 1). Dupilumab improved endoscopic features vs PBO at W24, as assessed by the EREFS total score (LSMD [95% CI], –3.07 [–4.26, –1.88]; –3.75 [–4.68, –2.82]; all *P*<0.0001) and inflammatory subscore (–2.80 [–3.86, –1.75]; –2.77 [–3.42, –2.11]; all *P*<0.0001), regardless of endoscopic profile. EREFS fibrostenotic subscore improved in pts with evidence of fibrostenotic disease at baseline (mixed profile) vs PBO at W24 (–0.29 [–0.60, 0.02], *P*=0.0660; –0.96 [–1.41, –0.52], *P*<0.0001).

**Conclusions:**

Regardless of the endoscopic profile, dupilumab leads to improvements vs PBO in histologic, symptomatic, and endoscopic EoE aspects in adults and adolescents with EoE at W24, with further treatment benefits at W52.

**Funding Agencies:**

Sanofi and Regeneron Pharmaceuticals Inc.